# Supplementary material for: Paternal exposure to benzo(a)pyrene induces genome-wide mutations in mouse offspring
Source: Commun Biol. 2019 Jun 20;2:228. doi: 10.1038/s42003-019-0476-5 (PMC6586636; doi:10.1038/s42003-019-0476-5)
Supplement: Supplementary file 1 — Supplementary Information [file 42003_2019_476_MOESM1_ESM.pdf]

Supplementary Discussion

Germline and Somatic Mosaic CNVs

Mosaicism, the presence of genetically unique cell populations in an organism, is recognized increasingly as a contributing source for genetic disease[1,2]. Mosaicism is produced from mutations that arise post-zygotically and are distributed to different tissues in various proportions. We investigated variants that were apparent germline mosaic CNVs arising post-zygotically in parents (unrelated to BaP exposure) and passed onto affected siblings. We observed two instances of germline mosaic large deletions in the mitotic BaP exposure group (Supplementary Data 6). The first CNV was present in two male littermates, and the second was present in three female littermates. The parental origin of the deletions could not be determined due to the lack of SNPs within the deletions. However, each deletion only appeared in one of the sires' four litters from separate dams (Supplementary Figure 5), suggesting that the deletion was maternal in origin. Interestingly, these deletions had a different mutation signature at the breakpoint than the *de novo* CNVs. Instead of microhomology at the breakpoint, there was a single nucleotide insertion (Supplementary Figure 6), which is characteristic of non-homologous end-joining. This difference is intriguing and suggests that further investigations to determine if there are different mechanisms by which CNVs are generated during spermatogenesis and oogenesis are warranted.

In addition to germline mosaics, we also observed a high number of somatic mosaic deletion events in the offspring (Supplementary Data 6). Based on quantitative PCR (qPCR) and aCGH alone, the somatic deletions originally appeared to be *de novo* events with a copy number of 1.19-1.37. However, mate-pair sequencing showed the presence of both parental alleles within

the deleted loci suggesting that these deletions occurred post-zygotically, and were, accordingly, somatic mosaics. The number of somatic events is likely higher than we observed because of the degree of stringency of the algorithms and filters used to call CNVs. For example, there were 3 additional deletions confirmed to have a copy number of 1.5-1.6 by qPCR that were eliminated by filtering (results not shown). Single-cell analyses will be required to measure the true extent of CNV mosaicism and to further determine the contribution of environmental influences during *in utero* development.

References

1. Zlotogora J. Germ line mosaicism. *Hum Genet* **102**, 381-386 (1998).

2. Campbell IM*, et al.* Parental somatic mosaicism is underrecognized and influences recurrence risk of genomic disorders. *Am J Hum Genet* **95**, 173-182 (2014).

Supplementary Figures

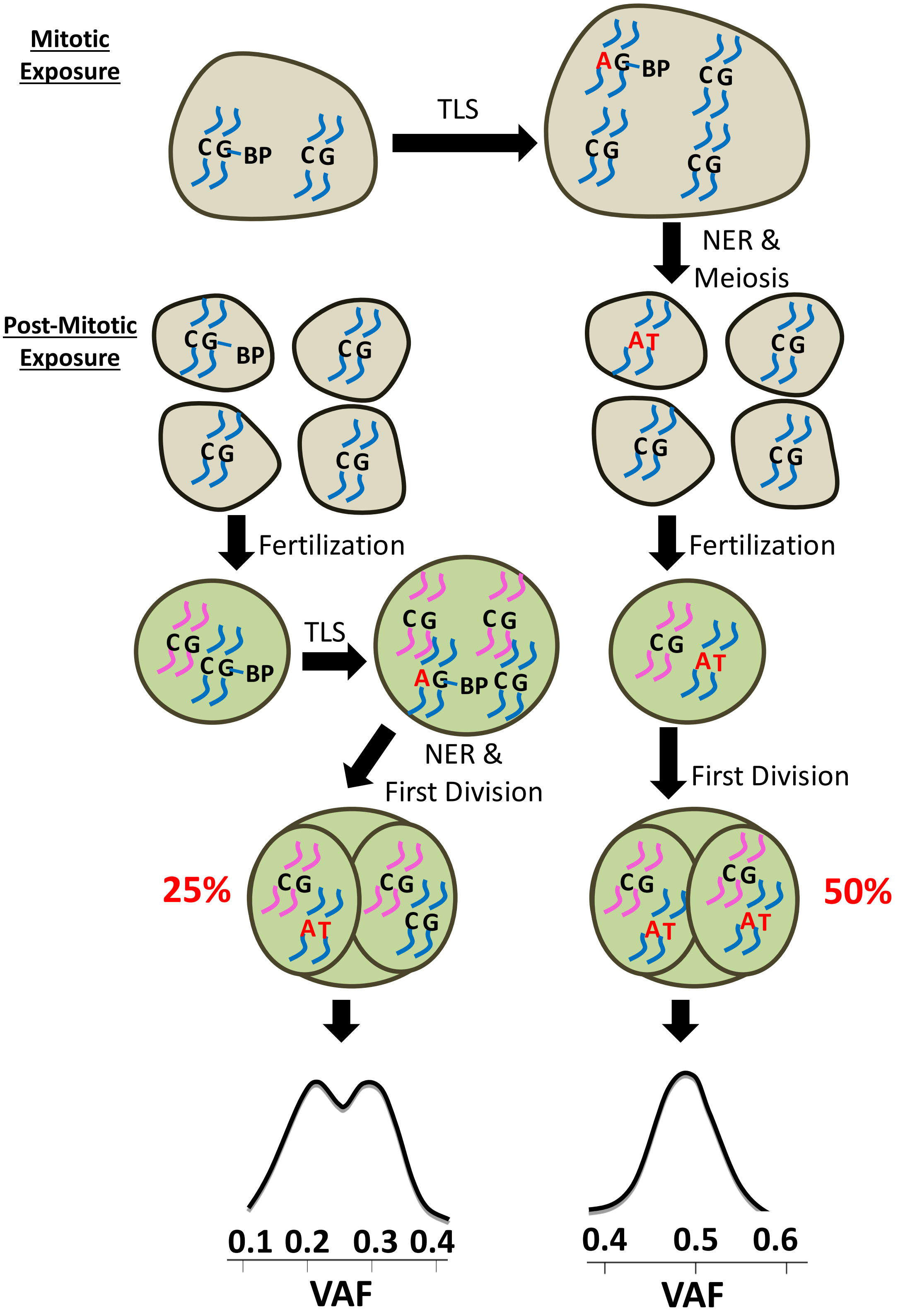

Supplementary Figure 1 | Mechanisms by which DNA adducts become embryonic or *de novo* mutations in the offspring. A mitotic exposure to BaP can lead to BPDE adducts (G-BP) in dividing spermatogonia. Translesion synthesis (TLS) results in error-prone replication and mismatch pairing. On the right, nucleotide excision repair (NER) will remove the bulky adduct and subsequent replication will lead to a mutation. If the sperm carrying that mutation fertilizes the egg the resulting embryo will have a paternal mutation with a VAF of 0.5. This matches the VAFs for *de novo* mutations detected in the offspring (bottom density plot). On the left, a new adduct is induced post-meiosis and the sperm carrying the adduct can fertilize the egg. Similar to dividing spermatogonia, TLS and NER in the embryo will convert the adduct to a mutation. Due to the semi-conservative nature of replication, the resulting embryo will have both the mutation and wild-type sequence from the sire. Therefore, the VAF for the mutation will be 0.25 in the early embryo. However, previous studies have shown that only three cells from the inner cell mass of the embryo go on to form the entire animal [30], resulting in VAFs of approximately 0.2, 0.3, or 0.5 in the animal. This is consistent with the VAFs for embryonic mutations detected in the offspring (as shown in density plot).

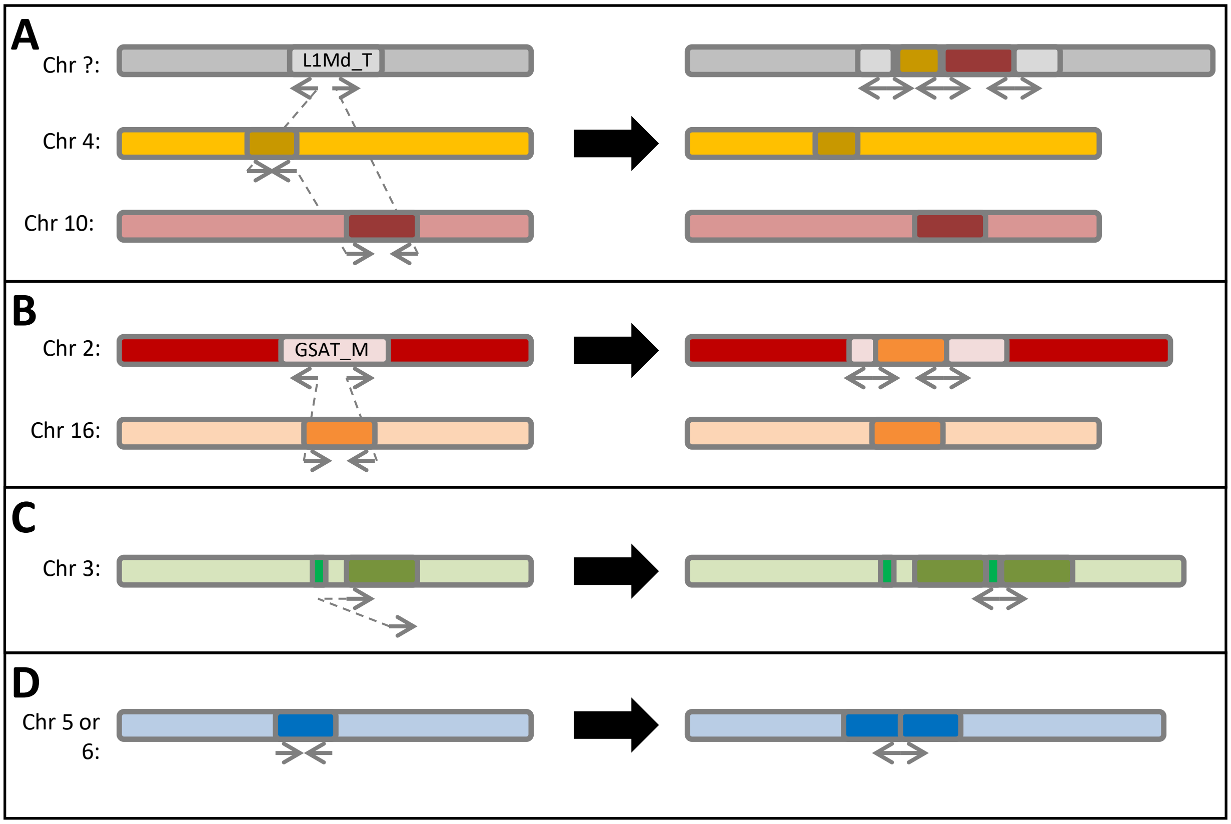

Supplementary Figure 2 | Overview of insertions and duplications detected in this study. The left side shows how the chromosomes appear in the reference genome (not to scale). The grey arrows indicate discordant mate pairs and the dashed lines indicate their partners. Using this information, the CNV structure could be identified (right side) with the mate pairs shown in the proper orientation. A) Sections of chromosomes 4 and 10 were inserted into a large L1Md_T element at an unidentified location in the genome. B) A segment of chromosome was inserted into a GSAT_M element, likely on chromosome 2. C) A tandem duplication occurred on chromosome 3. The breakpoint contained DNA from a 28bp region 16 kb upstream of the duplication. D) Two simple tandem duplications occurred in this study, 1 animal had the duplication on chromosome 5 and the other had it on chromosome 6.

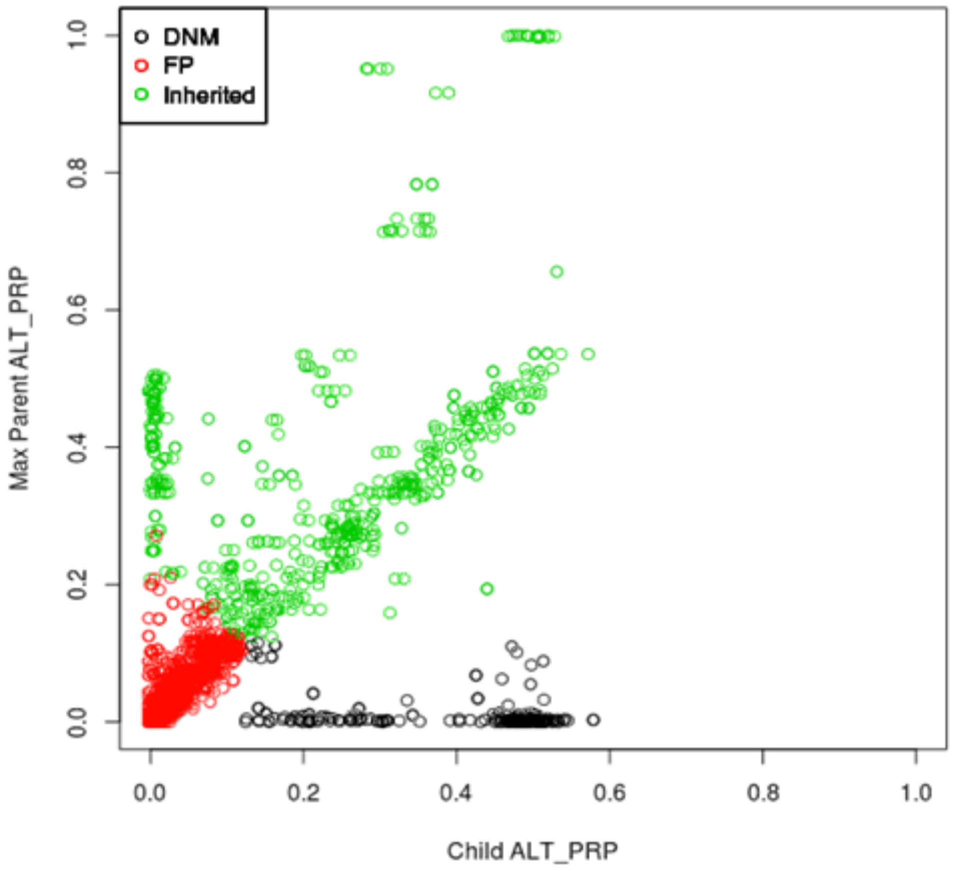

Supplementary Figure 3 | Validation of variants as *de novo* mutations, false-positives, or inherited. Each circle represents a variant call in an offspring. The Y-axis shows the maximum parental VAF for the variant and the X-axis shows the VAF in the offspring. Variants were called *de novo* here if the variant allele fraction was below 15% in parents and above 15% in offspring. The mutations were further filtered to eliminate mosaic and embryonic events.

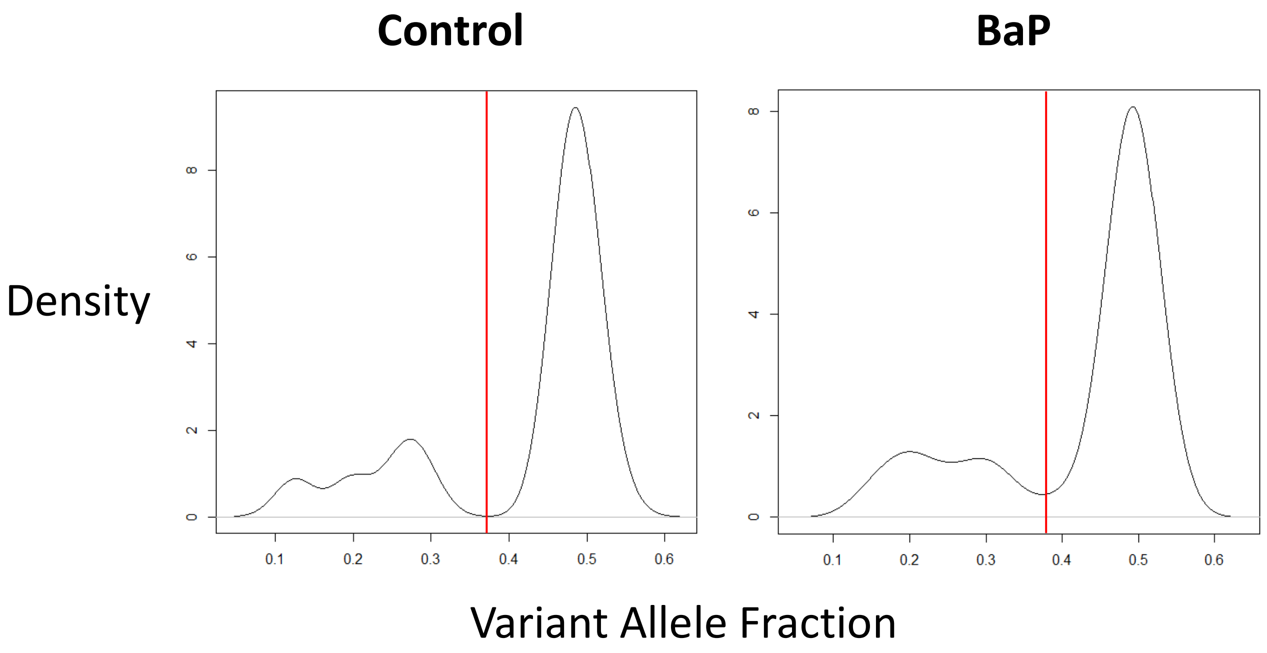

Supplementary Figure 4 | Density plots of variant allele fractions reveal 2 modes: embryonic mutations and *de novo* mutations. The red line (VAF = 0.39) indicates the threshold separating embryonic mutations from *de novo* mutations.

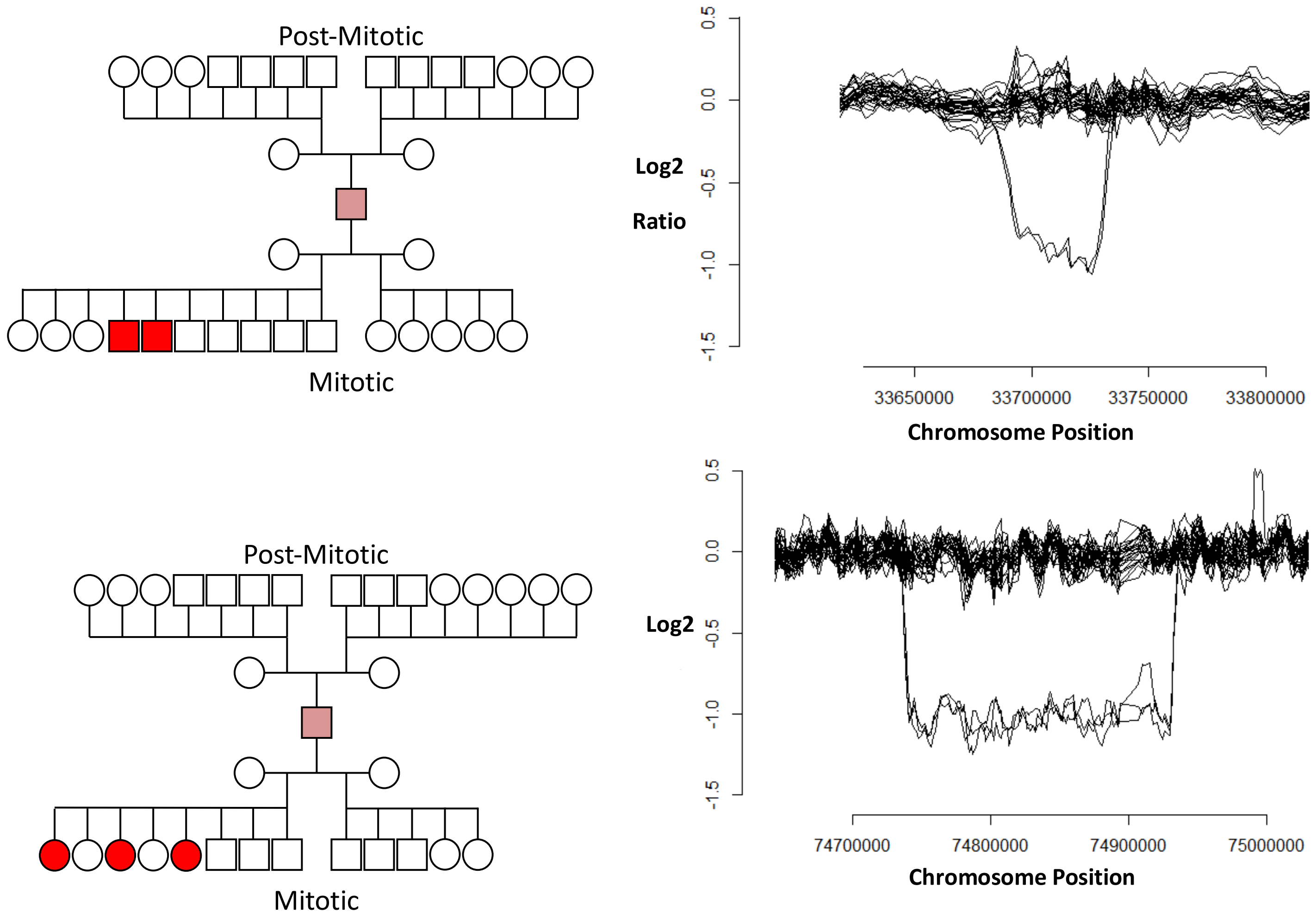
Post-Mitotic
Mitotic
Log2
Ratio
0.5
0.0
-0.5
-1.0
-1.5
33650000
33700000
33750000
33800000
Chromosome Position
Post-Mitotic
Mitotic
Log2
0.5
0.0
-0.5
-1.0
-1.5
74700000
74800000
74900000
75000000
Chromosome Position

Supplementary Figure 5 | Germline mosaic CNVs detected in the BaP exposure group (unrelated to exposure). The pink square indicates the sire that was exposed to BaP. The bright red circles and squares indicate female and male offspring that inherited the CNV. The parental origin of the mosaic CNVs could not be determined because there were no unique SNPs in the locus. The right panel indicates the log 2 signal intensities for probes across the chromosomal location of the CNV for unaffected and affected mice.

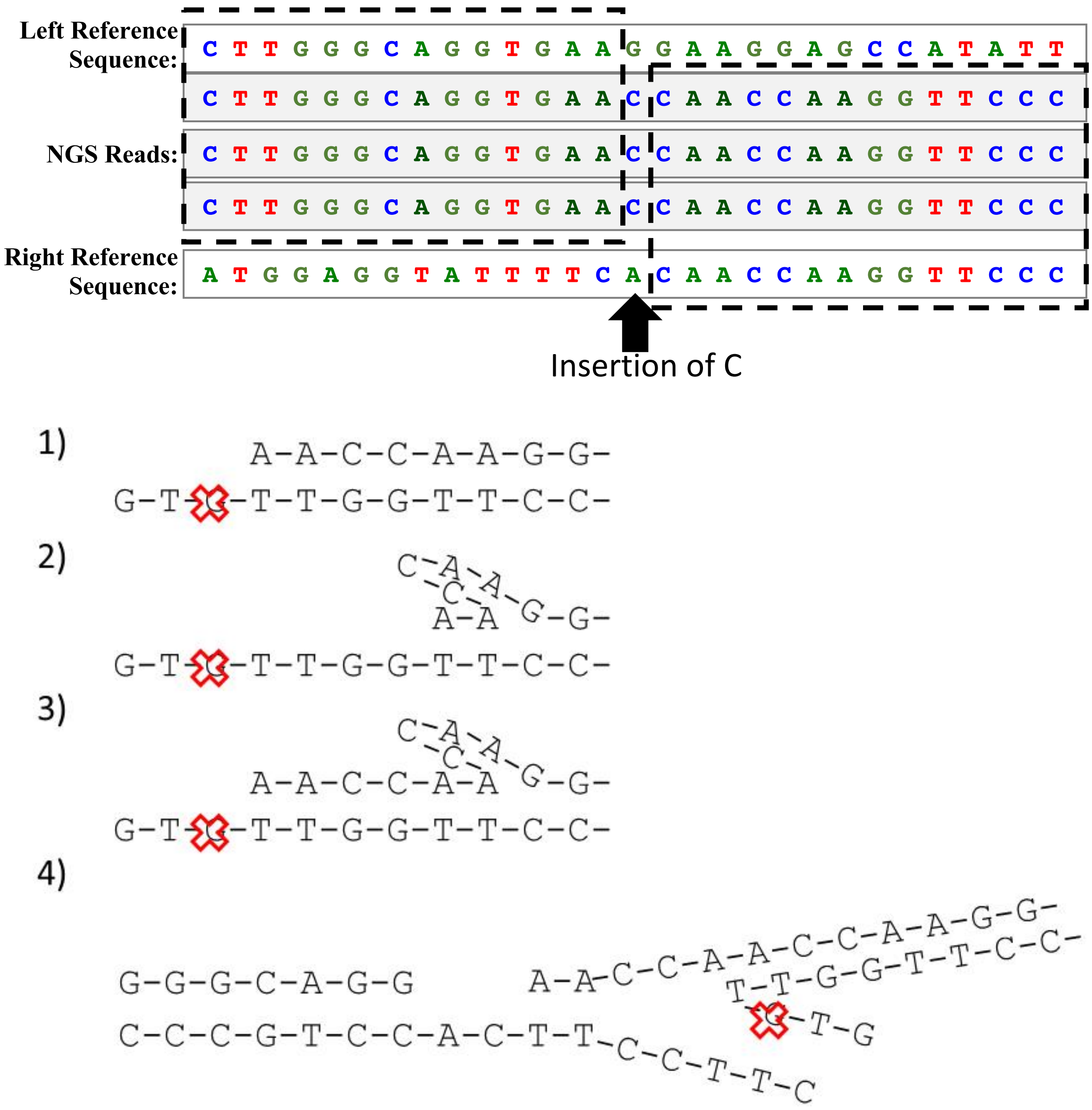

Supplementary Figure 6 | Different possible mechanisms that explain the germline mosaic CNV in animals 33FF1, 33FF3, and 33FF5. The top panel shows how the NGS reads align to the reference genome on the left and right sides of the CNV. The Cytosine nucleotide does not match either break sequence and may represent an insertion through Non-Homologous End-Joining. The bottom panel demonstrates a scenario where replication slippage can explain the mutation formation. (1) Replicating daughter strand pauses at damaged nucleotide. (2) Replication strand slips and re-anneals at incorrect template. (3) Replication re-initiates and duplicates sequence. (4) Failing to proceed on the template, the strand slips again and invades another replication fork downstream, using the microhomology of AA, resulting in a 196 kb deletion.
