# Supplementary material for: Paternal exposure to benzo(a)pyrene induces genome-wide mutations in mouse offspring
Source: Commun Biol. 2019 Jun 20;2:228. doi: 10.1038/s42003-019-0476-5 (PMC6586636; doi:10.1038/s42003-019-0476-5)
Supplement: Supplementary file 2 — Description of Additional Supplementary Files [file 42003_2019_476_MOESM2_ESM.docx]

**Supplementary Data**

**Supplementary Data 1 |** Overview of mutation candidates and detailed summary of *de novo* mutations detected in this study.

**Supplementary Data 2 |** Summary of embryonic mutations detected in this study.

**Supplementary Data 3 |** Consequences of mutations detected in this study.

**Supplementary Data 4** | Parental origin of SNVs with haplotype information.

**Supplementary Data 5** | Summary of *de novo* mutations and their locations.

**Supplementary Data 6 |** Detailed summary of CNVs detected in this study.

**Supplementary Data 7 |** Number of offspring analyzed by sire for the presence of copy number variants (CNV).

**Supplementary Data 8 |** Source data for Figure 2.

**Supplementary Data 9 |** Source data for Figure 4.
